# The border between progenitor cell recruitment and nephron shaping in the fetal human kidney during late gestation: a basic but understudied region

**DOI:** 10.3389/fneph.2025.1719394

**Published:** 2026-02-03

**Authors:** Will W. Minuth

**Affiliations:** Institute of Anatomy, University of Regensburg, Regensburg, Germany

**Keywords:** fetal human kidney, impairment of nephrogenesis, initial nephron shaping, nephrogenic compartment, nephrogenic zone, progenitor cell recruitment

## Abstract

**Introduction:**

The last 3 months of pregnancy are formative for the development of the fetal human kidney. Clinical experience with preterm and low birth weight infants indicates particular vulnerability during this period, as different noxae can terminate the development of new nephrons. This leads to oligonephropathy, which is associated with serious health consequences later in life. While the clinical aspects have been intensely investigated, few data point to the traces left by these noxae. In the nephrogenic zone, a reduction in its width and the absence of S-shaped bodies have been reported.

**Purpose:**

Not only the targets of noxae but also the site and early links of nephron formation remain poorly investigated. This concerns the individual compartments of the nephrogenic zone and the border between the district of progenitor cell recruitment (DPCR) and the area of nephron shaping (ANS) as a potential target of noxae.

**Methods:**

To shed initial light on this issue, the border between the DPCR and ANS was recorded using microanatomical criteria.

**Results:**

Nephron morphogenesis is shown to start at the far end of a cap mesenchyme, with the condensate opposite the head of a collecting duct (CD) ampulla still located within the DPCR. Driven by the mesenchymal-to-epithelial transition, the pretubular aggregate also arises at this site. Its proximal end remains adjacent to the connecting tubule of a previously formed nephron. Subsequently, it converts into the primitive renal vesicle, which thereafter expands within the ANS. Although separation is incomplete, the medial part of the distal pole adheres to the CD ampulla at the section border between its head and conus. This linkage of the future connecting tubule suggests that the conus of the CD ampulla co-elongates with the medial aspect of the shaping nephron stages.

**Conclusions:**

Closely related key points during early nephron formation are identified. Damage to only one of these points may result in either developmental arrest. Knowledge of these structural details is vital for pathological screening, interpretation of cell biological labeling, and identification of imprints left by noxae.

## 1 Introduction

The organogenesis of the human kidney is in various respects remarkable. However, clinically most relevant, and therefore especially challenging, are the last 3 months of pregnancy. These are formative, since during this period more than half of the total number of nephrons arises (1). Counting of the glomeruli revealed that the average number of nephrons in an adult human kidney is approximately 1 million, but there is considerable variation. Depending on internal and external conditions, it can range from as low as 200,000 to as high as 2.7 million (2, 3). In this context, it was registered that a low nephron endowment, which is reflected by a low starting nephron number, increases susceptibility to renal stress and chronic kidney disease (4, 5).

The experience with preterm and low birth weight infants indicates that fetal kidneys are especially vulnerable in the last months of pregnancy (6). It was recognized that a series of quite different noxae, such as malnutrition, diabetes, maternal vitamin deficiency, placental insufficiency, hyperoxia, as well as drugs, can evoke termination of nephrogenesis (7). This again leads to oligonephropathy, which is associated with severe health consequences later in life (8).

The numerous data raised in the clinical environment with preterm and low birth weight infants stand in contrast to a very limited understanding of the cellular and molecular targets of the mentioned noxae. Specifically, only a few pathological findings point to initial damage in the outer cortex of the fetal human kidney. For the externally situated nephrogenic zone, loss of basophilic S-shaped bodies was reported (9). Further, it was shown that in gestational controls the vertical width of the nephrogenic zone is not more than 150 µm, while in the group of preterm infants it is significantly smaller, at approximately 100 µm (10). However, definitive information addressing the related structural changes was not communicated. For the subjacent maturation zone, a reduced number and the occurrence of atypical glomeruli, which exhibit an extended Bowman’s space and a shrunken glomerular tuft, were described (11).

There are several reasons why few detailed structural data are available regarding impairment of nephrogenesis. One of them is that the microanatomical peculiarities of nephron formation in the fetal human kidney during advanced pregnancy were little noticed for a long time. Recently, the typical features of evolving glomeruli in the fetal human kidney from mid to late gestation were described (12). Additionally, the morphological specifics of the nephrogenic zone (13, 14), the shaping of transient nephron stages (15), mutual patterning with structural neighbors (16), and the different parts of the local interstitium (17) are all very important.

The microanatomical investigations performed in recent years have shown that the development of a nephron in the fetal human kidney during advanced pregnancy must be regarded as a process consisting of multiple links and taking place within different structural domains. At the start, it depends on recruitment of nephrogenic progenitor cells at the far end of the cap mesenchyme (18), but it is then subject to the successively running process of nephron shaping (15). This is mirrored by the arising of transient stages of the nephron anlage, such as the condensate, the pretubular aggregate, the different forms of renal vesicle stages, and the comma- and S-shaped bodies (19, 20). Very typical for the fetal human kidney, development of the later stages is restricted to the area of nephron shaping as an individual part of the nephrogenic zone (14). Furthermore, the increase in size of the nephron stages is accompanied by a series of changing shapes. In parallel, numerous interactions with respective structural neighbors, such as the related collecting duct ampulla or the local interstitium—including the vertically lining perforating radiate artery—occur (17).

It is important to recognize that nephron development is not an autarchic operation, but a highly interactive process. Installation of the shaping nephron is an essential part of it (21). This coincides with radial expansion of the parenchyma (15) and the interstitium (17). Since both tissues determine the vertical width of the nephrogenic zone in the fetal human kidney during advanced pregnancy, the structural changes occurring during expansion are of particular interest (22). Numerous data were generated dealing with availability, competence, and induction of nephrogenic progenitor cells (18). The initial shaping of the nephron was also communicated (15). However, surprisingly little explored is the interjacent transition that occurs when the district of progenitor cell recruitment is left and development continues with nephron shaping. To obtain information about the structural constellation and related key points at this border, a microanatomical analysis was performed. This represents a first approximation to a complex issue. Nevertheless, knowledge of these microanatomical details is indispensable for correct interpretation of data from high-throughput single-cell analyses or assessment of pathological specimens when initial traces left by noxae are investigated (23). In addition, a further aim of the present work is to create a structural basis so that, in subsequent steps, the identified key points can be used in digital image platforms to generate objective metrics for statistical comparisons.

## 2 Methods

### 2.1 Kidney preparation

To obtain a morphological view of the nephrogenic zone and the b9order between progenitor cell recruitment and nephron shaping, focus must be placed on the position of the collecting duct ampullae terminating at this site and on the transient stages of nephron anlage, such as the cap mesenchyme including the condensate, the pretubular aggregate, the renal vesicle stages, and the comma- and S-shaped bodies (15). In the fetal human kidney during advanced pregnancy, the nephrogenic zone is arranged as a thin strip along the inner side of the renal capsule. Consequently, damage to this particular region must be prevented during histological preparation. Therefore, a fetal human kidney was principally held at its hilum so that contact with the renal capsule by fine forceps was avoided. For the present investigation, 10 kidneys were analyzed.

### 2.2 Histological section plain

For microscopic analysis of histological sections, comparable perspectives are required (13). To achieve this, a fixed kidney was cut using a microtome from the renal capsule toward the papilla of a lobe. Following this approach, the section plane runs along the axis of the vertically oriented collecting duct (CD) tubules and their related ampullae, while at the same time being perpendicular to the renal capsule.

### 2.3 Selection of specimens

For the illustrations shown here, specimens of fetal human kidneys with a gestational age between weeks 16, 18, and 25 were selected from the stock of preparations used for the Course of Microscopic Anatomy for Medical Students at the University of Regensburg, Regensburg, Germany. These stages of kidney development are of particular interest, since during the last 3 months of pregnancy the majority of nephrons are formed in the outer cortex of the developing organ (1). Furthermore, the size of these organ stages is such that each kidney can be embedded as a whole for histology, thereby avoiding contact with or excision of the outer cortex. For the present investigation, specimens from gestational week 18 are shown.

### 2.4 Sample preparation

The tissue blocks were fixed in paraformaldehyde solution and embedded in paraffin wax according to routine methods. Sections of 5 µm thickness were then cut and stained with hematoxylin–eosin for analysis by optical microscopy. Screening of the stained sections was performed using a Leica DM750 microscope (Leica Microsystems, Wetzlar, Germany). Structural details were analyzed with an HI Plan 63x/0.75 objective lens. Images were captured using a Basler Microscopy Pulse 5.0 camera (Basler AG, Ahrensburg, Germany). All screened specimens exhibited a normal microanatomical appearance and good histological preservation. Causes of death, congenital abnormalities, renal cysts, or other pathological alterations were not observed.

### 2.5 Screening of specimens

More than 3000 images were available, which had been analyzed in earlier investigations dealing with nephron shaping (15), mutual patterning of the nephron with its structural neighbors (16), and the interjacent interstitium (17). From each analytical group, five representative images were selected that illustrate the border between nephrogenic stemness and shape generation and/or show the relationship between transient stages of nephron anlage and the collecting duct ampulla. Subsequently, one of these typical images was chosen for the illustrations shown here. To obtain metric information from the microscopic images and to insert the necessary labels, the software CorelDRAW 2021 (Corel Corporation, Munich, Germany) was used. The mean of at least three measurements is reported. It should be emphasized that the metric parameters presented here represent initial values intended for general orientation. Future studies should incorporate additional morphometric data obtained from healthy kidneys before comparisons with pathological specimens are undertaken.

### 2.6 Interpretation of images

**Figure 1:**
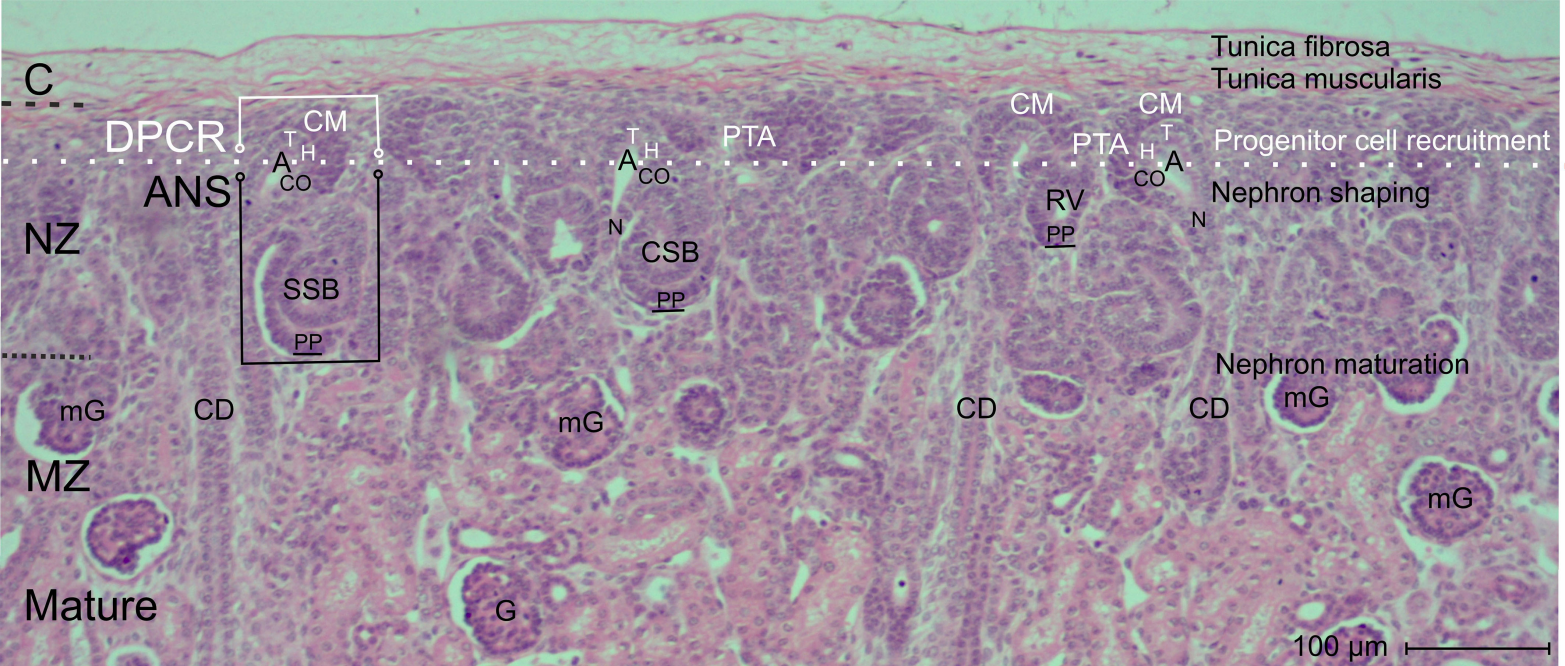
View of the outer cortex of the fetal human kidney during advanced pregnancy by optical microscopy. The nephrogenic zone (NZ) at gestational week 18 appears as a thin strip between the inner side (dashed black line) of the renal capsule (C) and the maturation zone (MZ; dotted black line). In the NZ, the cap mesenchyme (CM) and pretubular aggregate (PTA) face the tip (T) and head (H), respectively, of the related collecting duct (CD) ampulla (A). Beneath these, differently advanced transient stages of nephron anlage—such as renal vesicle (RV) stages, comma-shaped bodies (CSB), and S-shaped bodies (SSB)—are visible and positioned opposite the conus (CO) and neck (N) of a CD ampulla. Each of these stages develops within a nephrogenic compartment (white/black frame), consisting of the overlying district of progenitor cell recruitment (DPCR, white frame) and the subjacent area of nephron shaping (ANS, black frame). Both regions are separated by a transverse border (dashed white line). Structures in the DPCR are indicated in white, while components in the ANS are labeled in black. G, mature glomerulus; mG, maturing glomerulus; PP, proximal pole of the respective stage of nephron anlage.

**Figure 2:**
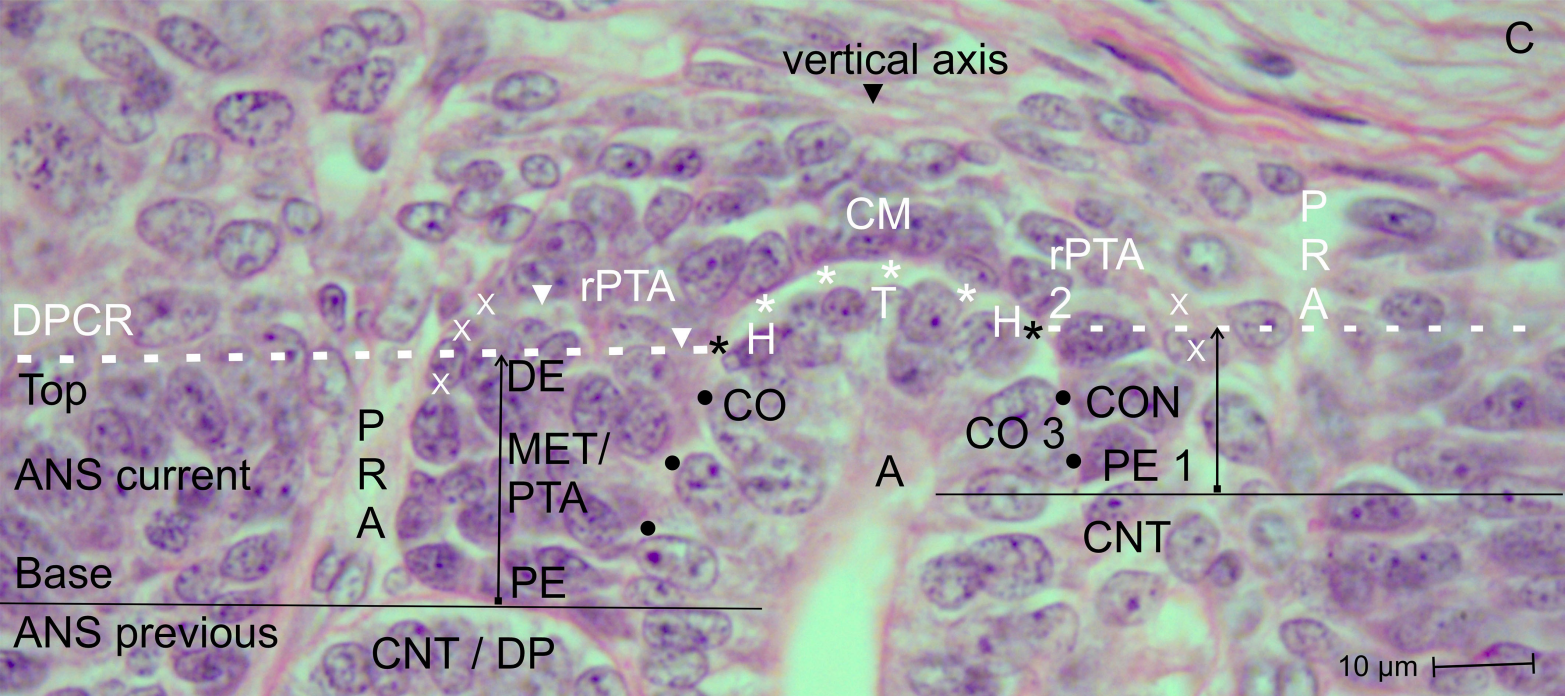
View of the nephrogenic zone of the fetal human kidney during advanced pregnancy (gestational week 18) by optical microscopy. The focus is directed toward the border (dashed white line) between the overlying district of progenitor cell recruitment (DPCR) and the subjacent area of nephron shaping (ANS). The border meets the endings (black asterisks) of the clear interface (white asterisks) located between the inner side of the cap mesenchyme (CM) and the tip (T) and head (H) of the CD ampulla (A). On the right side of the image, the far end of a cap condensate (CON) faces the border. The proximal end (PE) of the condensate is positioned next to the connecting tubule of a previously formed S-shaped body. Its medial side (black dots) adheres to the conus (CO) of the CD ampulla. On the left side of the image, an earlier-initiated nephron anlage undergoes the mesenchymal-to-epithelial transition to form a pretubular aggregate (PTA). Its proximal end (PE) remains adjacent to the connecting tubule (CNT) of a previously developed nephron. At its distal end (DE), incomplete separation (arrowhead) occurs, resulting in separation of only the medial side from the overlying residual pretubular aggregate (rPTA). In contrast, the lateral side remains connected (white X). Vertical arrows indicate ongoing radial expansion. A triad of key points is identified: (1) Anchoring of the proximal end next to the connecting tubule of a previously formed nephron; (2) incomplete transverse separation at the cap condensate; and (3) linkage at the conus of the CD ampulla. C, renal capsule; PRA, perforating radiate artery.

**Figure 3:**
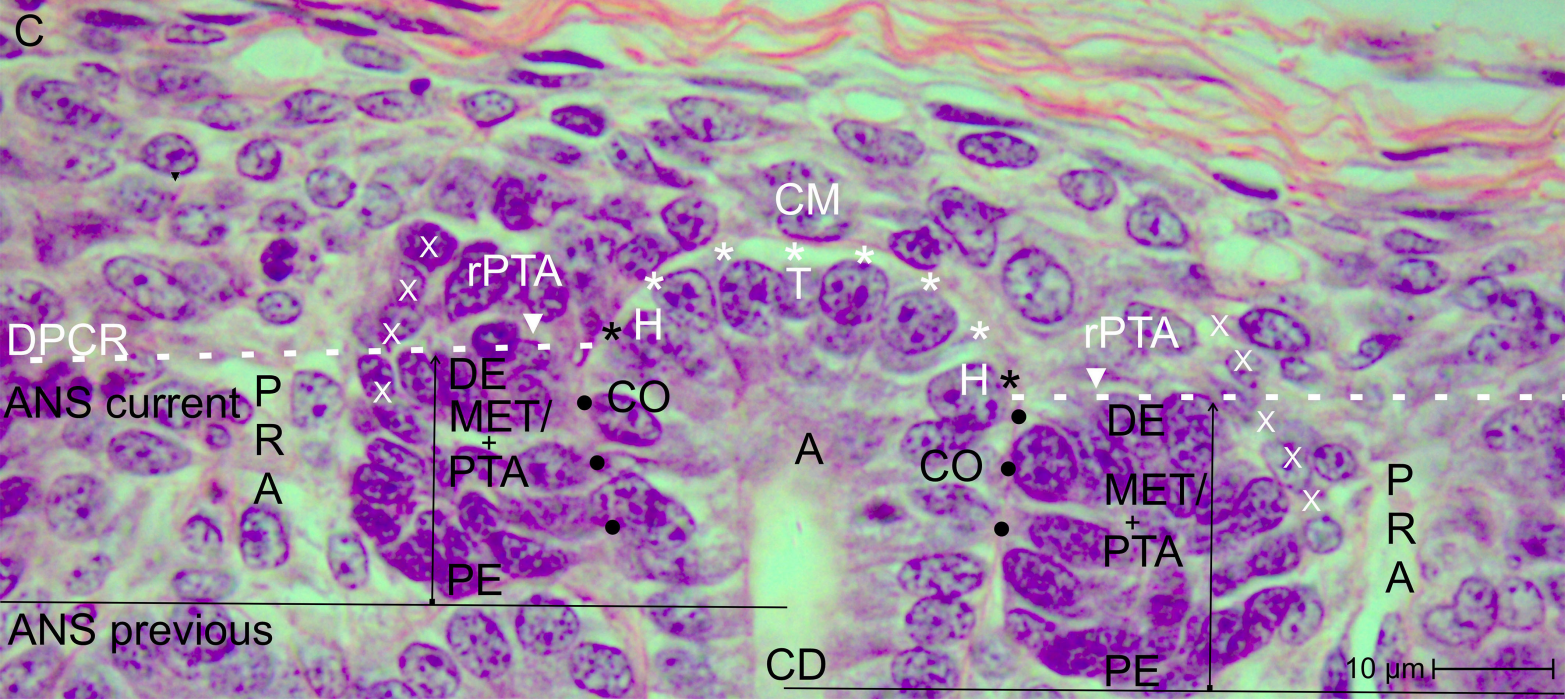
View of the nephrogenic zone of the fetal human kidney during advanced pregnancy (gestational week 18) by optical microscopy, focusing on the border (dashed white line) between the overlying district of progenitor cell recruitment (DPCR) and the subjacent area of nephron shaping (ANS) during formation of the pretubular aggregate (PTA). This process is driven by the mesenchymal-to-epithelial transition (MET). The border meets the endings (black asterisks) of the clear interface (white asterisks), which is located between the inner side of the cap mesenchyme (CM) and the tip (T) and head (H) of the CD ampulla (A). In this example, pretubular aggregates are visible on both sides of the CD ampulla. At the proximal end (PE), formation of a faint basal lamina is observed, while a primary lumen (black cross) appears centrally. At the distal end (DE), incomplete separation (arrowhead) is evident, resulting in persistence of a lateral connection via a progenitor cell strand (white X). The medial side of the developing pretubular aggregate shows adhesion (black dots) to the conus (CO) of the CD ampulla. Vertical arrows indicate ongoing radial expansion. C, renal capsule; PRA, perforating radiate artery.

**Figure 4:**
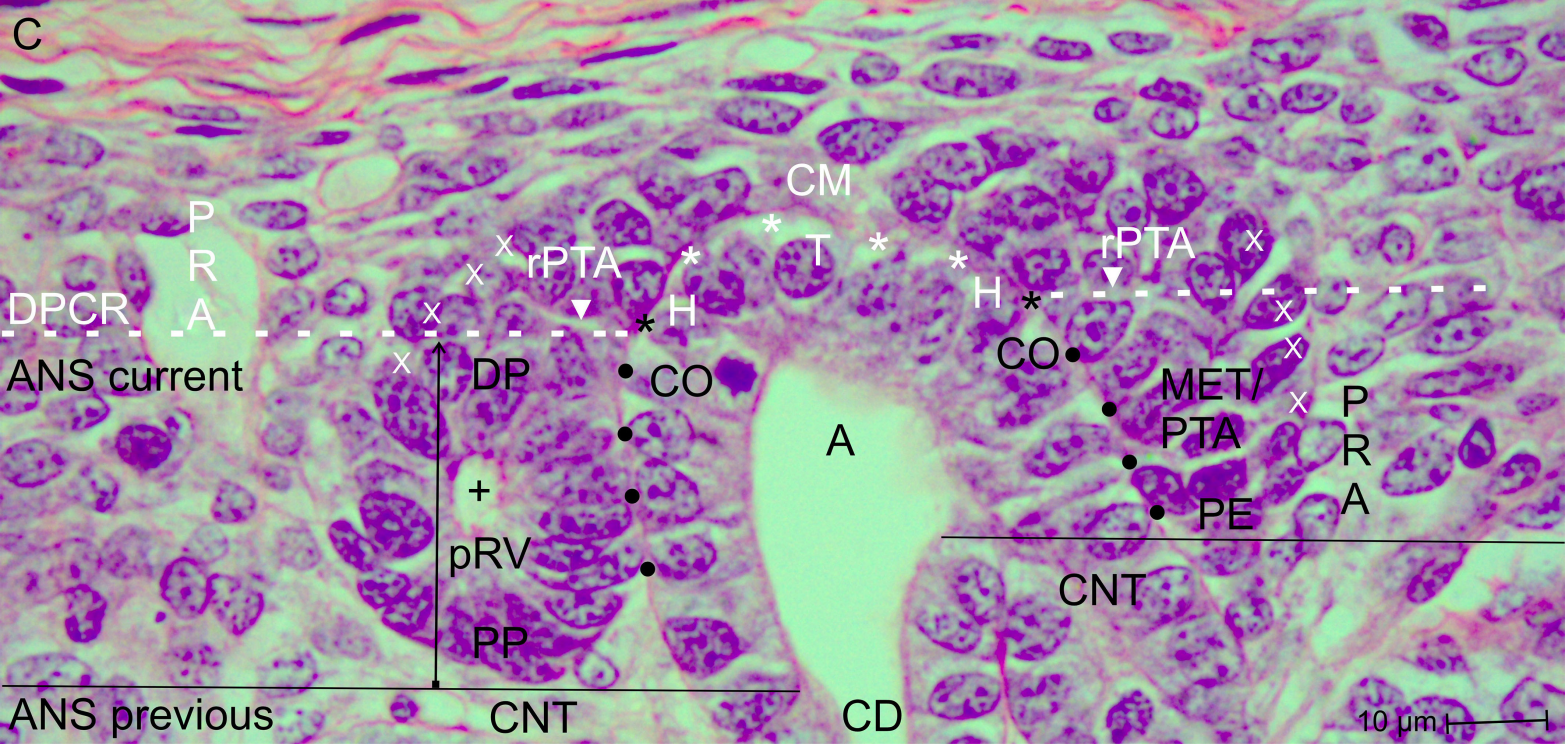
View of the nephrogenic zone of the fetal human kidney during advanced pregnancy (gestational week 18) by optical microscopy. The focus is directed to the border (dashed white line) between the overlying district of progenitor cell recruitment (DPCR) and the subjacent area of nephron shaping (ANS). On the left side of the image, formation of a primitive renal vesicle (pRV) occurs. Above the border, the residual pretubular aggregate (rPTA) is present at the ending (black asterisk) of the clear interface (white asterisks). It is incompletely separated (arrowhead) from the distal pole of the underlying primitive renal vesicle. The remaining progenitor cell strand (white X) serves temporary provision and vertically crosses the border. Further, the transversely running border meets the related collecting duct (CD) ampulla (A) at the border between its head (H) and conus (CO). In this situation, the medial aspect of the primitive renal vesicle shows a close relationship (black dots) to the conus of the CD ampulla. The proximal pole is positioned opposite the connecting tubule (CNT) of a previously formed nephron. The lateral aspect is exposed to the perivascular interstitium of a perforating radiate artery (PRA). On the right side of the image, a pretubular aggregate (PTA) arises, driven by the mesenchymal-to-epithelial transition (MET). At its lateral side, wide intercellular spaces are visible. +, primary lumen; C, renal capsule; CM, cap mesenchyme.

**Figure 5:**
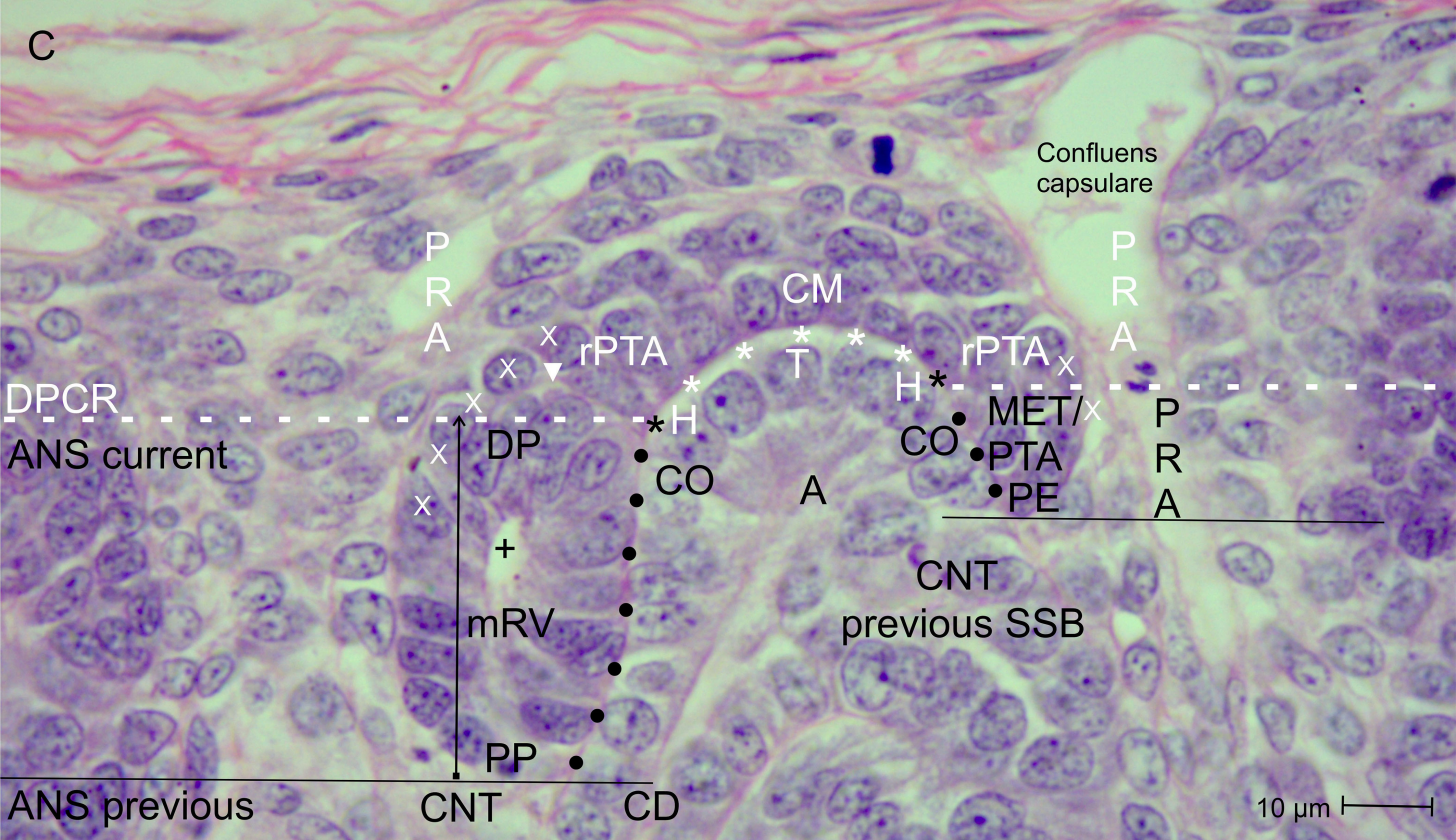
View of the nephrogenic zone of the fetal human kidney during advanced pregnancy (gestational week 18) by optical microscopy. The focus is directed to the border (dashed white line) between the overlying district of progenitor cell recruitment (DPCR) and the subjacent area of nephron shaping (ANS). The border meets the end (black asterisk) of the clear interface (white asterisks) near the section border between the head (H) and conus (CO) of the related collecting duct (CD) ampulla (A). On the right side of the image, the proximal end (PE) of an arising pretubular aggregate (PTA), driven by the starting mesenchymal-to-epithelial transition (MET), is fixed next to the connecting tubule (CNT) of a previously formed S-shaped body (SSB). Its medial side (black dots) contacts the conus (CO) of the CD ampulla. At its lateral side, the temporary connecting progenitor cell strand (white X) appears and is exposed to the perivascular interstitium of a perforating radiate artery (PRA). On the left side of the image, formation of a mature renal vesicle (mRV) takes place. Its proximal pole (PP) is positioned next to the connecting tubule (CNT) of a previously formed nephron. Between its medial aspect and the conus of the related CD ampulla, an apparent proximity (black dots) is observed. At its distal pole (DP), the future connecting tubule (CNT) is visible. Above, partial separation (arrowhead) from the residual pretubular aggregate (rPTA) occurs. This is delimited externally by the connecting progenitor cell strand (white X), which vertically crosses the border. The vertical arrow indicates the current progress of radial expansion. +, primary lumen; C, renal capsule; CM, cap mesenchyme.

**Figure 6:**
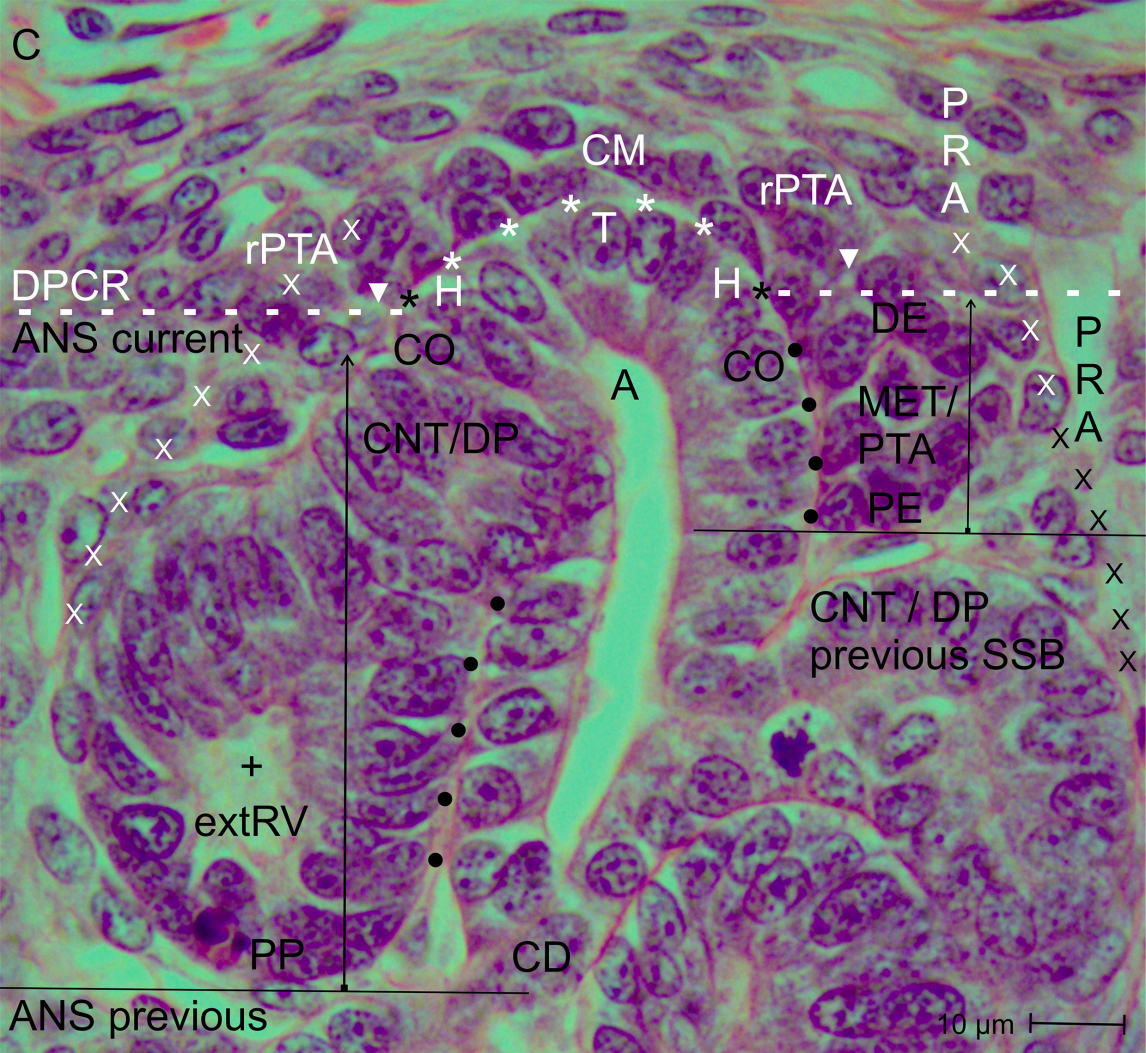
View of the nephrogenic zone of the fetal human kidney during advanced pregnancy (gestational week 18) by optical microscopy. The focus is directed to the border (dashed white line) between the overlying district of progenitor cell recruitment (DPCR) and the subjacent area of nephron shaping (ANS). The border meets the end (black asterisk) of the clear interface (white asterisks) and thereby points to the section border between the head (H) and conus (CO) of the related collecting duct (CD) ampulla (A). On the left side of the image, formation of an extending renal vesicle (extRV) occurs. Its proximal pole (PP) is positioned next to the connecting tubule (CNT) of a previously formed nephron. Its medial aspect and the conus of the CD ampulla exhibit an apparent proximity (black dots). At its distal pole (DP), the future connecting tubule (CNT) invades the CD ampulla. Adjacent to this site, partial separation (arrowhead) from the residual pretubular aggregate is visible. Externally, this is delimited by the temporary progenitor cell strand (white X), which vertically crosses the border. On the right side of the image, a pretubular aggregate (PTA) driven by the mesenchymal-to-epithelial transition (MET) arises. Its proximal end (PE) is clearly outlined and remains positioned opposite the connecting tubule (CNT) of the previously formed S-shaped body (SSB). At the lateral side of the pretubular aggregate, a further connecting progenitor cell strand (white X) with wide intercellular spaces develops. It vertically crosses the border and is exposed to the perivascular interstitium of a perforating radiate artery (PRA). The earlier temporary progenitor strand (black X) from the previous nephron generation has disappeared. The vertical arrow indicates the current progress of radial expansion. + primary lumen; C, renal capsule; PRA, perforating radiate artery; CM, cap mesenchyme.

**Figure 7:**
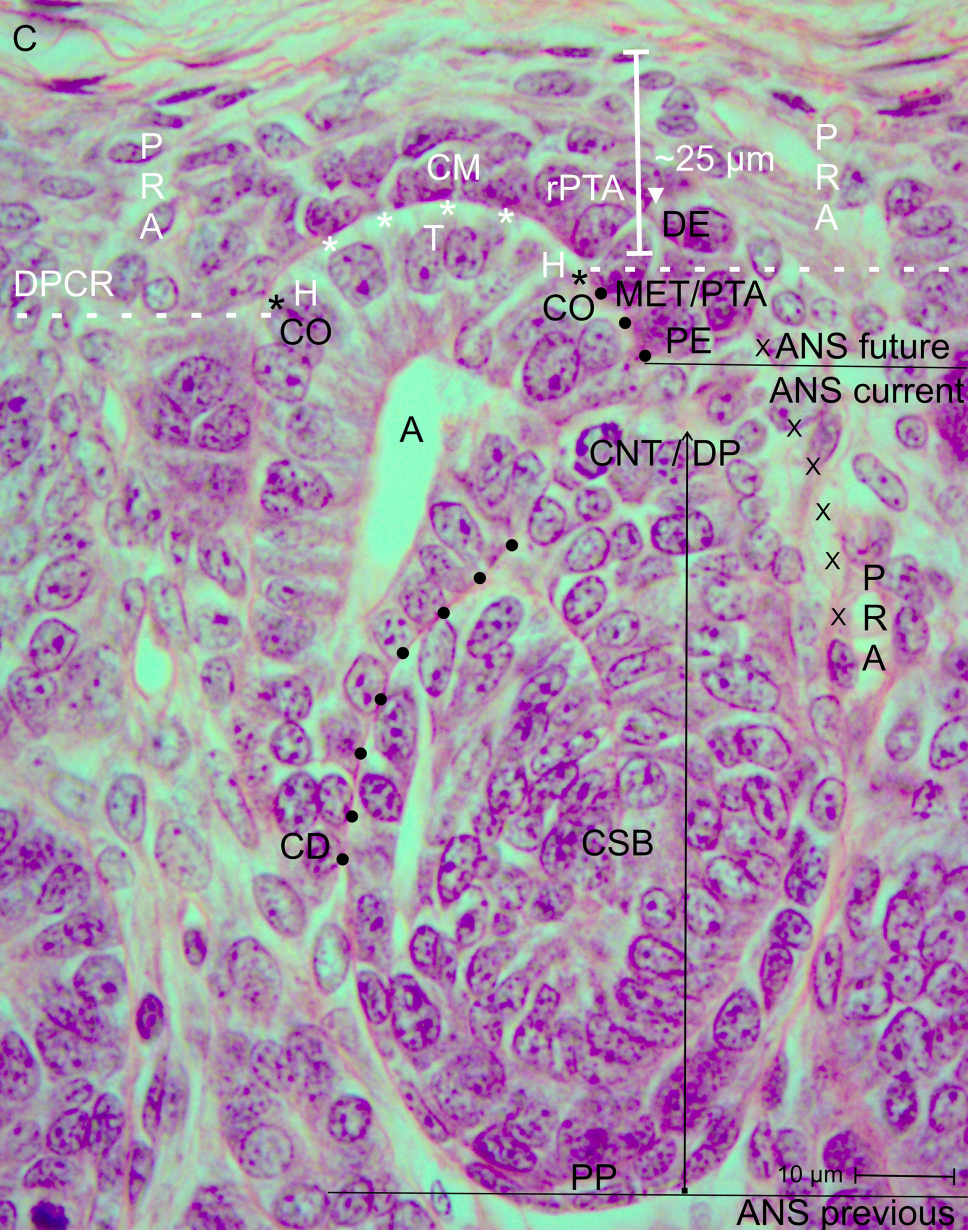
View of the nephrogenic zone of the fetal human kidney during advanced pregnancy (gestational week 18) by optical microscopy. The focus is directed to the border (dashed white line) between the overlying district of progenitor cell recruitment (DPCR) and the subjacent area of nephron shaping (ANS). The border meets the end (black asterisk) of the clear interface (white asterisks) and thereby points to the related collecting duct (CD) ampulla (A) between its head (H) and conus (CO). In the upper part of the image, the structural relationship between an arising pretubular aggregate (PTA), driven by the mesenchymal-to-epithelial transition (MET), and the underlying comma-shaped body (CSB) is demonstrated. The medial side of the proximal end of the arising PTA adheres (black dots) to the conus of the CD ampulla. The center of the proximal end of the PTA is positioned opposite the connecting tubule (CNT) of the formed comma-shaped body. At the lateral side, the previously present temporary progenitor cell strand (black X) is lost. This occurs near the perivascular interstitium of a perforating radiate artery (PRA). In the middle and lower part of the image, a comma-shaped body is visible. Its proximal pole (PP) is positioned at the connecting tubule (CNT) of a previously formed nephron. Between its medial aspect and the conus of the neighboring CD ampulla, a marked proximity (black dots) is observed. At its distal pole (DP), the connecting tubule (CNT) has invaded the CD ampulla. The vertical arrow indicates the current progress of radial expansion. + primary lumen; C, renal capsule; PRA, perforating radiate artery; CM, cap mesenchyme.

To visualize the morphological situation at the border between nephrogenic progenitor cell recruitment and nephron shaping, original images are presented. In the illustrations shown (**Figures 1**-**7**), structures in the district of progenitor cell recruitment are indicated in white, while the components in the area of nephron shaping are labeled in black. A transverse dashed white line indicates the interjacent border. Furthermore, the proximal pole (fix-point, lower side of the image) of the currently developing nephron anlage rests adjacent to the connecting tubule of a previously developed nephron. Since the space at this site is limited, the corresponding distal pole (mobile-point, upper side of the image) of the developing nephron anlage shifts radially in concert with the elongating conus of the collecting duct (CD) ampulla during ongoing development.

## 3 Results

When the outer cortex of the fetal human kidney during advanced pregnancy is examined at low magnification using optical microscopy, a line can be drawn parallel to the inner side of the renal capsule (**Figure 1**; **Table 1**). Above this line lies the part of the nephrogenic zone associated with progenitor cell recruitment. Below this line, the shaping nephron stages, such as the renal vesicles, the comma-shaped bodies, and the S-shaped bodies, are positioned. Since concrete information regarding this transition is not available, the involved structures are described below.

**Table 1:** List of the different functional claims in the outer cortex of the fetal human kidney during advanced pregnancy.

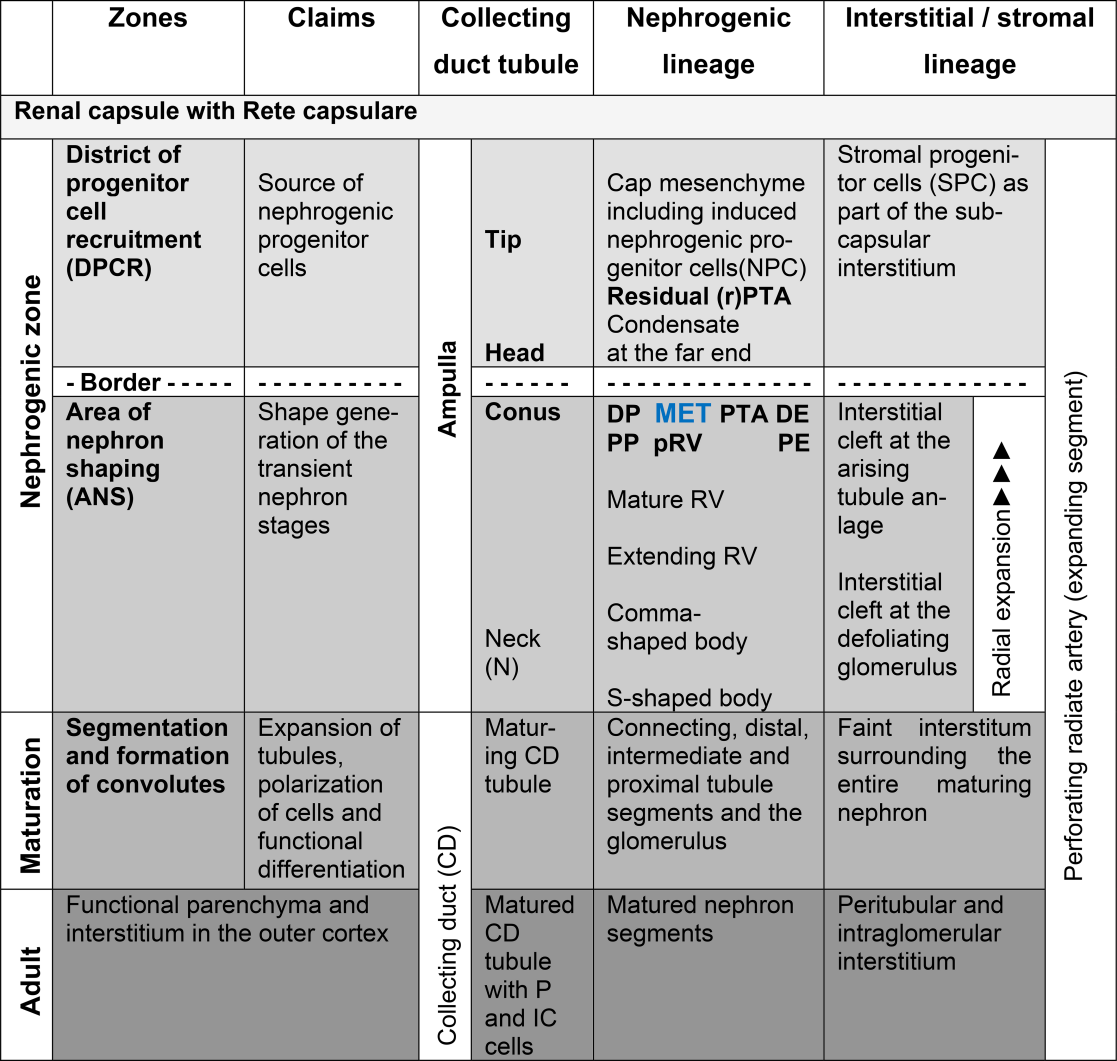

### 3.1 Nephrogenic zone

Images obtained by optical microscopy reveal that the nephrogenic zone is recognized as a thin strip of evolving parenchyma and interstitium (Latin)/stroma (Greek) (**Figure 1**). Without showing a sharp contrast, its external side faces the tunica muscularis of the externally situated renal capsule, while the internal side of the nephrogenic zone continues into the subjacent maturation zone.

Typical structural components of the nephrogenic zone are the vertically aligned collecting duct (CD) tubules. These exhibit ureteric bud–derived CD ampullae at their endings (**Figure 1**). The tip of each ampulla is covered by the cap mesenchyme, including competent nephrogenic progenitor cells. Laterally and opposite the head of a CD ampulla, at the far end of the cap mesenchyme, the condensate and the resulting pretubular aggregate are positioned. Beneath this, and strictly allocated to the conus of a CD ampulla, the medial aspect of either a renal vesicle stage, a comma-shaped body, or an S-shaped body is observed. Between these structures, the vertically oriented perforating radiate arteries and the local interstitium occur.

Microscopic screening of the nephrogenic zone further shows that shaping nephron stages progressively increase in size (**Figure 1**). This influences the local vertical width of the nephrogenic zone, which is determined by measuring the distance between the inner side of the renal capsule and the proximal pole of the respective nephron stage. When nephron stages at different developmental levels are present, the vertical width of the nephrogenic zone varies from site to site. If the respective proximal poles are connected, a transverse but graduated line is obtained, separating the overlying nephrogenic zone from the underlying maturation zone. In contrast, between the distal end of the separating pretubular aggregates and the corresponding residual part of the pretubular aggregate at the cap mesenchyme, a transverse but straight line can be drawn. This line indicates the border between nephrogenic progenitor cell recruitment above and nephron shaping below.

From a schematic point of view, the border between nephrogenic progenitor cell recruitment and nephron shaping appears as a straight line. In reality, however, it represents a small zone of evolving tissues. First, at the far end of the cap mesenchyme, the condensate arises and, restricted to this site, develops via the mesenchymal-to-epithelial transition (MET) into the pretubular aggregate and subsequently into the primitive renal vesicle. Second, at the section border between the head and conus of the CD ampulla, the conus radially elongates together with the medial aspect of the currently shaping nephron. Third, and most important for local microvascularization, is the corresponding section of a perforating radiate artery at the lateral aspect of the shaping nephron. Finally, indispensable is the interjacent and correspondingly extending interstitium.

### 3.2 Nephrogenic compartment

For the microanatomical reading of the nephrogenic zone, it is recommended to allocate each nephron stage to a nephrogenic compartment (**Table 2**). When this approach is adopted, it becomes evident that the nephrogenic compartments are aligned in a single row and arranged side by side. Within each compartment, the current state of development can be registered.

**Table 2:** List of prominent microanatomical features in the nephrogenic zone of the fetal human kidney during advanced pregnancy.

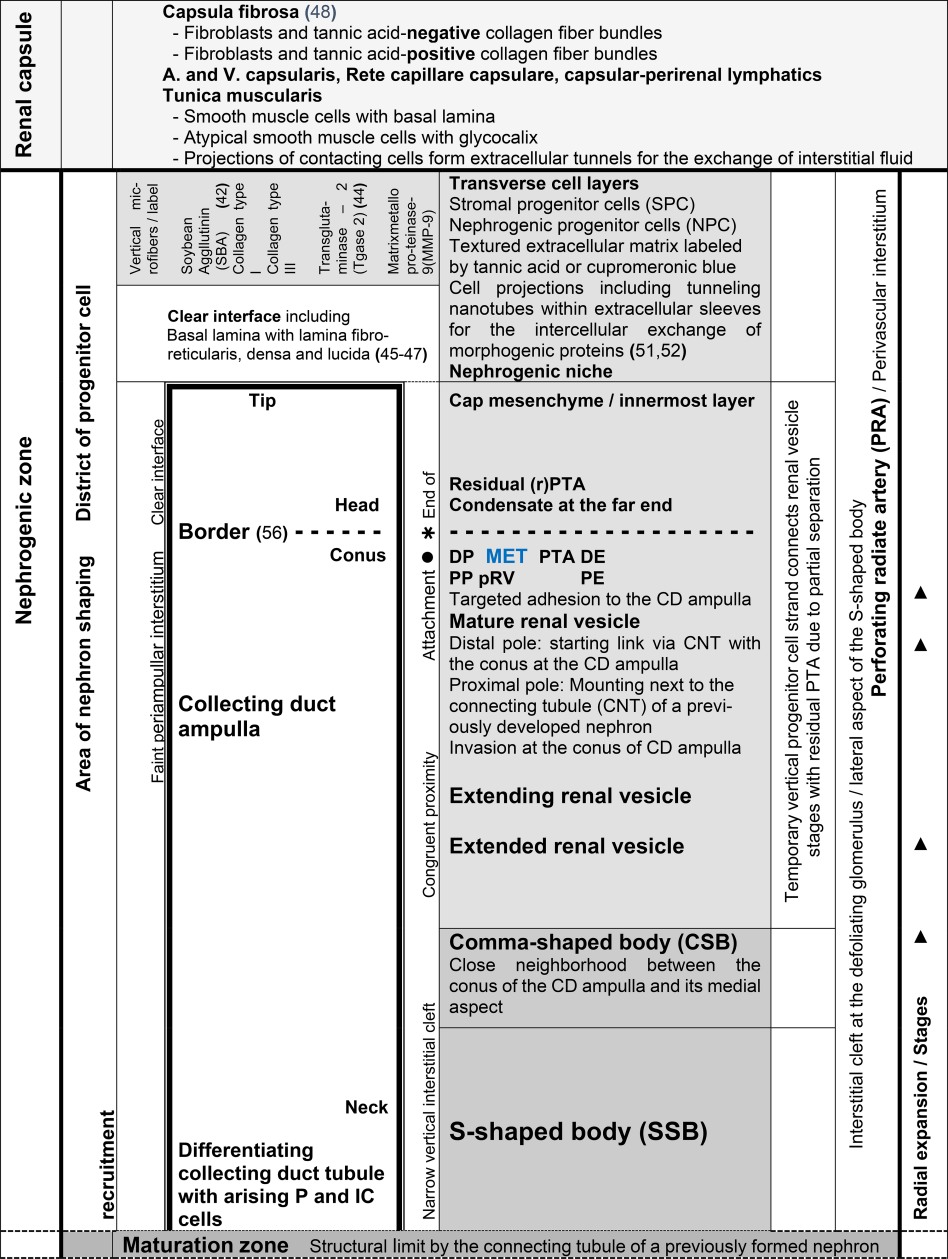

At the top, a nephrogenic compartment is covered by the renal capsule (**Figures 1**, **2**: **Table 2**). In the center, the collecting duct (CD) ampulla is recognized as a prominent structure. Separated by a clear interface, nephrogenic progenitor cells are contained at its tip within the cap mesenchyme. Somewhat laterally at its head, faint points of adhesion indicate structural interactions between the cap mesenchyme and the CD ampulla. At the section border between the head and conus, the condensate at the far end of the cap mesenchyme transforms via the mesenchymal-to-epithelial transition first into the pretubular aggregate and then into the primitive renal vesicle. The medial aspect of the subsequent renal vesicle stages, as well as the comma- and S-shaped bodies, faces the correspondingly elongating conus. Meanwhile, the respective lateral aspect of these stages is flanked by a developing perforating radiate artery. This vessel ascends from the maturation zone, passes through the nephrogenic zone, and joins the rete capsulare in the renal capsule. The base of a nephrogenic compartment faces the connecting tubule, which represents the distal pole of a previously formed S-shaped body.

When a transverse line is drawn along the distal end of a pretubular aggregate, it meets the section border between the head and conus of the related CD ampulla. In this way, the nephrogenic compartment is subdivided (**Figure 2**; **Table 2**). The space positioned above represents the durable district of progenitor cell recruitment. Beneath it, the repetitively opening area of nephron shaping is present. Consequently, the interjacent narrow cleft represents the border between progenitor cell recruitment and nephron shaping.

### 3.3 CD ampulla and its subdivision into sectors

The investigation shows that the initial development of a nephron—from nephrogenic progenitor cell recruitment up to the S-shaped body—occurs in close interaction with distinct sectors of the related ureteric bud–derived collecting duct (CD) ampulla (**Figures 2**-**7**). It is observed that this development does not start accidentally. Instead, the formation of the condensate (**Figure 2**), the pretubular aggregate (**Figure 3**), and the primitive renal vesicle (**Figure 4**) begins only when a previously formed and underlying comma-shaped body is completing its development. Meanwhile, the cap mesenchyme remains positioned between the inner side of the renal capsule and the tip of a CD ampulla (**Figure 4**). This space is stable and exhibits a vertical width of 15–20 µm. In this situation, the tunica muscularis of the renal capsule faces a thin layer of subcapsular interstitial progenitor cells. Beneath this, the cap mesenchyme is present in the form of only two layers of nephrogenic progenitor cells. Consequently, the innermost layer of nephrogenic progenitor cells is exposed to the basal aspect of epithelial progenitor cells integrated into the tip of the CD ampulla. This site represents the nephrogenic niche.

Further screening demonstrates that the CD ampulla is vertically oriented and composed of a monolayered polarized epithelium. While remaining in a constant position, its structural neighbors are consistently arranged in the same configuration. This observation suggests subdivision of the CD ampulla into distinct sectors (**Figures 3**, **4**). Accordingly, the blindly ending sector adjacent to the renal capsule represents the tip, while laterally the head of the CD ampulla is located. Directly opposite this region, the medial side of the condensate or, respectively, the pretubular aggregate is positioned.

Between the innermost layer of the cap mesenchyme (including competent nephrogenic progenitor cells) and the tip of the CD ampulla (including epithelial progenitor cells), a clear interface is observed (**Figures 2**, **3**). In contrast, between the head of the CD ampulla and the medial side of the condensate and the emerging pretubular aggregate, this clear interface terminates. This site corresponds to the section border between the stationary head and the elongating conus of the CD ampulla. Consequently, it marks the border between the overlying district of progenitor cell recruitment and the underlying area of nephron shaping (**Table 2**). Restricted to this site, the future connecting tubule of the shaping nephron stages becomes linked.

### 3.4 Initial position of the nephron

Opposite the tip of a collecting duct (CD) ampulla, competent nephrogenic progenitor cells located in the innermost layer of the cap mesenchyme are induced by morphogenic proteins. Meanwhile, these cells translocate laterally toward the head of the CD ampulla for assimilation at the far end of the cap mesenchyme (**Figures 2**, **3**). It can be observed that the condensate and the pretubular aggregate are restricted to this site. Consequently, the future proximal end of the pretubular aggregate remains positioned next to the connecting tubule of a previously developed, underlying S-shaped body. Closer inspection of the histological situation reveals, at the lateral side of the emerging pretubular aggregate, a row of cells (**Figure 4**, right side of the image). Between these cells, remarkably wide intercellular spaces are visible. This structure develops into the progenitor cell strand mentioned later, which temporarily connects progenitor cell recruitment with nephron shaping. In contrast, the medial side of the pretubular aggregate shows adhesion at the section border between the head and conus of the CD ampulla.

Of particular importance for histological screening, the proximal end at the condensate–pretubular aggregate marks the base position of the morphologically recognizable nephron (**Figure 2**, right side of the image; **Figure 3**; **Figure 4**, right side of the image). Originating from this position and remaining at this site, by the mesenchymal-to-epithelial transition, the circular arrangement of nephrogenic progenitor cells and, finally, the formation of the primitive renal vesicle take place (**Figure 4**, left side of the image). Since fundamental events of nephron positioning and assembly are concentrated here, the spatial surroundings are of particular interest. The lateral part of the proximal end of the pretubular aggregate is exposed to the perivascular interstitium of a vertically oriented perforating radiate artery. The central part of the proximal end is positioned adjacent to the connecting tubule of a previously developed nephron. The medial side of the pretubular aggregate adheres to the conus of the related CD ampulla. This arrangement indicates that the proximal end at the pretubular aggregate remains fixed, whereas, driven by radial development of the shaping nephron stages, the corresponding distal pole shifts in concert with the elongating conus of the CD ampulla.

### 3.5 Recruitment replaced by shape generation

A crucial step in the chain of nephron formation is the termination of progenitor cell recruitment, followed by shape generation of the nephron. This process is driven by the mesenchymal-to-epithelial transition (**Figures 2**-**4**). The determining site is the arising of the condensate at the far end of the cap mesenchyme. First, a discrete cell aggregation is observed (**Figure 2**, right side of the image). Then, a pre-epithelial clamp, which originates at the proximal end of the pretubular aggregate, is registered (**Figure 2**, left side of the image; **Figure 3**, left and right sides of the image). Within this structure, a primary lumen becomes visible. Finally, the primitive renal vesicle is present (**Figure 4**, left side of the image). Its lumen is enclosed by a single-layered polarized epithelium. At its basal side, an inconsistent basal lamina is observed.

One would expect complete separation of the renal vesicle from the overlying residual pretubular aggregate at the cap mesenchyme. However, the specimens show that this separation is, for the time being, incomplete. While the primitive renal vesicle (**Figure 4**) develops into the mature renal vesicle (**Figure 5**) and then into the extending renal vesicle (**Figure 6**), only the medial part of its distal pole exhibits transverse separation from the overlying residual pretubular aggregate. In contrast, the lateral part of the distal pole at the different renal vesicle stages remains connected.

The entire lateral aspect of the renal vesicle stages is exposed to the perivascular interstitium of an ascending perforating radiate artery. At the distal pole of these renal vesicle stages, the initial adhesion at the section border between the head and the upper end of the conus of the CD ampulla expands to become a true attachment, enabling successive invasion of the future connecting tubule. At the corresponding medial aspect, a remarkable proximity to the conus of the CD ampulla is observed. In this situation, the proximal pole of the renal vesicle stages remains positioned next to the connecting tubule of a previously formed nephron.

### 3.6 Opening-up of a radially expandable space

Structural development of the primitive (**Figure 4**), mature (**Figure 5**), and extending renal vesicle (**Figure 6**) stages, which is accompanied by incomplete separation from the overlying residual pretubular aggregate, requires a change in the surrounding structural context. One of the governing requirements is the need for space during shaping within the nephrogenic zone. Due to previously developed parenchymal and interstitial structures in the underlying maturation zone, such space is not available at the proximal pole of the currently shaping renal vesicle stages. At the lateral aspects, some expandable space exists. However, the greatest available space lies at the distal pole in the radial direction.

Closer examination reveals that the proximal pole of the renal vesicle stages represents a fix-point, which remains anchored as the base next to the connecting tubule of a previously formed nephron (**Figures 4**-**6**). In contrast, the distal pole represents the mobile-point, which is translocated during eccentric shaping in the radial direction. In **Figures 2**-**7**, this movement is indicated by thin vertical arrows. Accordingly, the increasing distance between the two poles reflects radial expansion of the currently shaping nephron stages. The distal pole thereby corresponds to the future connecting tubule. When a transverse line is drawn at this level, it indicates the base of a future area of nephron shaping. Above this line lies the border between the district of progenitor cell recruitment and the area of nephron shaping. Consequently, further nephron shaping occurs only beneath this border.

As a result of eccentric but radial expansion of the shaping nephron, the vertical parameters of the nephrogenic zone increase (**Figures 3**-**7**). A vertical distance of 10 µm is measured at the cap condensate located between the connecting tubule of a previously formed nephron and the head of the related CD ampulla (**Figure 2**, right side of the image). During the mesenchymal-to-epithelial transition, radial expansion of the pretubular aggregate increases to 25 µm (**Figure 2**, left side of the image; **Figure 3**). When the primitive renal vesicle forms, this distance increases to 35 µm (**Figure 4**). During development of the mature renal vesicle, radial expansion reaches up to 40 µm (**Figure 5**). At the onset of the extending renal vesicle stage, 60 µm are measured (**Figure 6**). During formation of the late comma-shaped body, radial expansion approaches 100 µm (**Figure 7**). A maximum of 110 µm is reached when the S-shaped body has formed. Importantly, while radial expansion proceeds, it lifts the overlying district of progenitor cell recruitment—including the renal capsule, the pool of nephrogenic progenitor cells in the cap mesenchyme, and the tip and head of the CD ampulla—en bloc, without disturbing the preexisting structural relationships.

### 3.7 Crossing structures at the border

The border between progenitor cell recruitment and nephron shaping contains vertically crossing structures. Most striking among these is the ureteric bud–derived CD ampulla (**Figures 1**, **7**). While its tip and head (**Figure 2**) remain positioned within the district of progenitor cell recruitment, its conus and neck (**Figures 3**-**7**) are consistently situated in the area of nephron shaping. Its shaft is located within the maturation zone.

The CD ampulla exhibits distinct regional peculiarities. As described above, the tip is separated from the facing nephrogenic progenitor cells by a clear interface (**Figures 2**-**7**). In contrast, between the head of the CD ampulla and the medial side of the pretubular aggregate, this clear interface terminates (**Figures 2**, **3**). At this site—corresponding to the section border between the head and conus of the CD ampulla—the initial adhesion of the arising nephron occurs. This represents the morphological sign of the initial linkage of the future connecting tubule to the henceforth shaping nephron. In addition, it marks the site at which the vertically aligned CD ampulla crosses the transverse border. Above this level, the head of the CD ampulla awaits the next interaction with the facing condensate located in the cap mesenchyme. At the section border between the head and conus of the CD ampulla, invasion of the future connecting tubule will take place. Below this level, within the newly opened area of nephron shaping, the conus of the CD ampulla expands together with the medial aspect of the currently shaping nephron (**Figures 4**-**7**).

A further crossing structure emerges during partial separation of the primitive renal vesicle (**Figure 4**). It remains visible at the mature renal vesicle (**Figure 5**) and extending renal vesicle stages (**Figure 6**), but degrades during formation of the comma-shaped body (**Figure 7**). This structure corresponds to the progenitor cell strand, which aligns from the lateral side of the overlying residual pretubular aggregate to the subjacent lateral aspect of the shaping nephron stages.

In addition to the CD ampulla and the transitional progenitor cell strand, the associated interstitium also crosses the border between progenitor cell recruitment and nephron shaping. Due to its continuous presence, it plays a role in formation of the condensate within the cap mesenchyme and structuring of the pretubular aggregate (**Figures 2**-**5**). Furthermore, it can be observed that, at the lateral side of the pretubular aggregate, the subcapsular interstitium meets the perivascular interstitium. This convergence is associated with a vertically ascending and, at the same time, progressively elongating perforating radiate artery (**Figures 6**, **7**).

## 4 Discussion

Three decades ago, the theory of fetal programming of tissues manifesting in the adult organism was formulated by J. P. Barker and C. Osmond (24). It proposes that poor maternal living conditions during pregnancy provide the basis for the onset of diseases later in life. An example supporting this concept is the vulnerability of the fetal human kidney during advanced pregnancy, since quite different kinds of noxae can terminate the formation of new nephrons (25, 26). It has been shown that this leads to oligonephropathy with severe health consequences later in life (27).

Suspected targets of noxae within the nephrogenic zone include the progenitor cells (28, 29), shaping of the transient nephron stages up to the S-shaped body (9), branching and elongation of the ureteric bud–derived collecting duct ampullae (30, 31), the linkage between the collecting duct and the connecting tubule (32, 33), and completion of the interjacent interstitium, including the vital microvascular supply (17, 34) (**Table 1**; **Figures 1**, **2**). However, to date, none of these noxae have had their precise cellular or molecular targets identified.

### 4.1 Installation rail for a developing nephron

In the fetal human kidney, the majority of nephrons is formed during the last trimester of pregnancy (1). Initially, this coincides with radial expansion of the parenchyma and interstitium within the nephrogenic zone (22). Over time, this process is reflected in organ growth, which is driven by proliferative activity in the shaping nephrons, the related CD ampullae, and the local interstitium (35). Surprisingly, this growth does not occur in a synchronous manner, but rather in a site- and demand-specific fashion. Consequently, the generated data indicate that this task is managed by single, self-sufficiently operating nephrogenic compartments (**Figure 1**; **Tables 1**, **2**).

The logistical processes related to provision of nephrogenic progenitor cells are located within the overlying district of progenitor cell recruitment (**Figures 2**, **3**; **Tables 1**, **2**) (19). In contrast, processes associated with initial nephron shaping occur in the underlying area of nephron shaping. To initiate this process, nephrogenic progenitor cells within the cap mesenchyme that face the tip of a CD ampulla acquire competence for induction by morphogenic proteins (18, 36). Subsequently, these progenitor cells translocate to the far end of the cap mesenchyme to initiate formation of a condensate opposite the head of the CD ampulla. Through the mesenchymal-to-epithelial transition, this structure transforms into the pretubular aggregate (37). Restricted to the same site, subsequent development of the primitive renal vesicle takes place (**Figure 4**), which then transforms through eccentric but radial expansion into the mature (**Figure 5**) and extending (**Figure 6**) renal vesicle stages, the comma-shaped body (**Figure 7**), and finally the S-shaped body (15, 22).

### 4.2 Above the border

A closer examination of a nephrogenic compartment in the fetal human kidney during late gestation shows that the structural composition of its externally situated district of progenitor cell recruitment is highly specific (**Table 2**; **Figures 2**-**4**). The space between the inner side of the renal capsule, the tip of a collecting duct (CD) ampulla, and the interjacent cap mesenchyme is distinctive (13). Here, interstitial progenitor cells, nephrogenic progenitor cells, and epithelial progenitor cells are densely arranged (29, 38, 39). Restricted to the innermost cell layer of the cap mesenchyme, nephrogenic progenitor cells interact with the subjacent epithelial progenitor cells, which are integrated into the tip of a CD ampulla, enabling reciprocal exchange of morphogenic proteins (40). Astonishingly, although both progenitor cell types cooperate, they are maintained at a distance by a clear interface (**Figures 2**–4) (41).

Immunohistochemical studies revealed that the nephrogenic progenitor cell layers opposite the tip of a CD ampulla are traversed by microfibers, which can be labeled, for example, with soybean lectin (SBA) (42) or antibodies recognizing collagen type III, a typical component of the interstitial matrix (43). In the neonatal rabbit kidney, it was further demonstrated that these microfibers are both static and dynamic, since extracellular matrix–stabilizing tissue transglutaminase 2 (Tgase2) and matrix-degrading metalloproteinase 9 (MMP9) are co-expressed at this site (**Table 2**) (44). Also noteworthy are the ultrastructural features of the clear interface at the tip of a CD ampulla (45–48) and the extracellular matrix glycoproteins present there (49, 50). Particularly striking are projections of nephrogenic progenitor cells that cross the clear interface within sleeves of extracellular matrix to link with the basal aspect of epithelial progenitor cells (51, 52). This arrangement most likely serves targeted exchange of poorly soluble morphogenic proteins. Interestingly, the data presented here confirm earlier investigations demonstrating direct cell-to-cell contacts within the nephrogenic niche of the mouse kidney (53) and in transfilter culture experiments (54). To what extent this complex structural configuration is controlled by extracellular matrix mechanics remains to be clarified (55). Apart from detection of strong labeling with the cell proliferation marker Ki-67 (35), comparable cell biological data for the area of nephron shaping are not yet available.

### 4.3 Mesenchymal-to-epithelial transition at the border

Earlier labeling experiments with tannic acid in the neonatal rabbit kidney revealed that a sharp border exists between the extracellular matrix of progenitor cell recruitment and that of nephron shaping (56). The present investigation of the fetal human kidney indicates that this transversely aligned border represents a transition between tissues at different developmental stages located above and below. According to microanatomical criteria, it also represents the interface between the basal side of the overlying district of progenitor cell recruitment and the apical side of the underlying area of nephron shaping, which fulfill distinct morphological and physiological roles (**Table 2**; **Figure 2**). Consequently, the data generated here identify key structures that define the interjacent border as a critical site for these little-explored developmental processes. These include promotion of the condensate at the far end of the cap mesenchyme, the mesenchymal-to-epithelial transition occurring at this site, and subsequent formation of the pretubular aggregate in the same location (57–60).

The associated microanatomical key points are of particular importance. One fundamental key point is termination of the clear interface facing the head of a CD ampulla (**Figures 2**, **3**). Another key point is adhesion of the emerging renal vesicle to the upper conus of the CD ampulla (**Figure 4**) (61). This adhesion appears essential not only for positioning but also for subsequent expansion of the renal vesicle (62). An additional key point is the partial transverse separation between the residual pretubular aggregate and the medial part of the distal pole of the subjacent renal vesicle (**Figures 4**, 5). When a line is drawn from this key point toward the CD ampulla, it points to the concealed transverse section border between its head and conus (**Figure 6**).

A particularly striking but poorly explored feature is the progenitor cell strand spanning between the residual pretubular aggregate and the lateral end of the distal pole of the various renal vesicle stages (**Figures 4**-**6**). Whether this structure serves provision of additional imprinted nephrogenic progenitor cells remains unknown. As the comma-shaped body forms, this strand disappears (**Figure 7**).

### 4.4 Shaping underneath the border

The present investigation indicates that the space beneath the border separating progenitor cell recruitment from nephron shaping functions through a temporary opening-up. Restricted to this region, development of the transient stages of nephron anlage—from the pretubular aggregate (**Figures 2**, **3**) to the S-shaped body—takes place (15). Depending on the developmental progress within a nephrogenic compartment, the available space is minimal when a pretubular aggregate (**Figure 3**) or a primitive renal vesicle (**Figure 4**) is present. During the eccentric development of the mature (**Figure 5**) and extending (**Figure 6**) renal vesicle stages, as well as the comma- (**Figure 7**) and S-shaped bodies, the respective spaces increase. However, due to a previously formed and underlying nephron, expansion is not possible at the proximal pole, which represents the base of the currently shaping nephron. Instead, expansion proceeds at the distal pole in a radial direction. Correspondingly, this process lifts the border between progenitor cell recruitment and nephron shaping, so that the overlying district of progenitor cell recruitment is translocated en bloc while preserving its structural composition.

The obtained data further demonstrate a determining linkage between the connecting tubule of the currently shaping nephron and the upper part of the conus of the CD ampulla (**Figures 4**-**7**). This linkage causes the conus of the CD ampulla to elongate consistently in concert with the medial aspect of the radially expanding nephron stages (21).

### 4.5 Structured search for the initial traces left by noxae

This work demonstrates that the nephrogenic zone in the fetal human kidney during advanced pregnancy is characterized by a complex microarchitecture (**Figures 1**-**7**). A central role is played by the border between progenitor cell recruitment and nephron shaping. Most likely, this border is particularly vulnerable and represents a primary target of noxae impairing nephrogenesis.

Earlier studies showed that noxae of diverse molecular origin—such as malnutrition, maternal diabetes, vitamin deficiency, placental insufficiency, hyperoxia, and drugs—can evoke termination of nephrogenesis in the fetal human kidney during advanced pregnancy (7). One might expect that each noxa induces a distinct pattern of histological damage. However, for none of the noxae has an individual damage pattern, a specific cellular or molecular target, or a related metabolic process been identified to date. For this reason, it is essential to determine whether there is a shared process within the nephrogenic zone of the fetal human kidney during advanced pregnancy that is affected by all noxae. Potential candidates include the Hippo pathway (63), cellular metabolic pathways altered by diabetes (64), and/or reduced expression of morphogenic proteins such as Bmp7, Gdnf, and WT1 (26).

The present investigation provides new data on the initial formation of a nephron, which may serve as potential targets of noxae. Of particular importance is the transition from progenitor cell recruitment through the mesenchymal-to-epithelial transition to initial nephron shaping. It is demonstrated that, within each nephrogenic compartment of the nephrogenic zone, sequential spaces exist that differ in structural composition and in which site-specific developmental steps occur (**Figures 2**-**7**; **Tables 2**, **3**). First, this finding enables selection of appropriate analytical tools for a systematic search for initial traces left by noxae. Second, based on the structural conditions demonstrated here, specific search fields can be defined to increase the likelihood of detecting suspected traces left by noxae (65). Specifically, the search fields for clarifying the causes of loss of basophilic S-shaped bodies (8) include the district of progenitor cell recruitment, the area of nephron shaping, and in particular the interjacent border (**Figures 2**, **3**). To identify causes of reduced vertical width of the nephrogenic zone in preterm infants (9), analysis must focus on radial elongation of the conus of the CD ampulla, which proceeds in concert with the medial aspect of transient nephron stages restricted to the area of nephron shaping (22). In contrast, to obtain information regarding occurrence of atypical glomeruli exhibiting an extended Bowman’s space and a shrunken glomerular tuft (11), investigation should be directed toward the maturation zone.

**Table 3:** List of the different functional claims in the outer cortex of the fetal human kidney during advanced pregnancy.

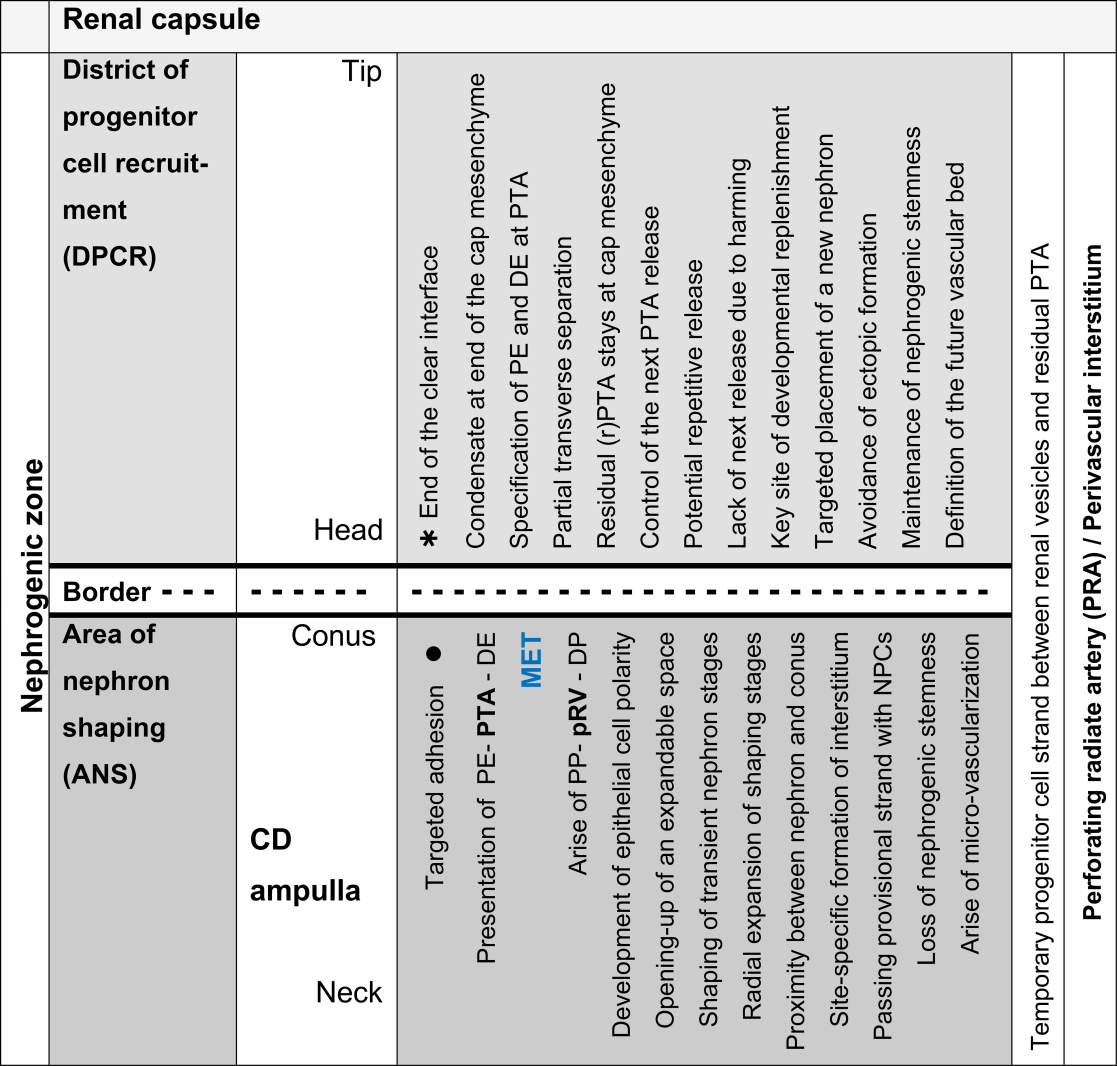

The generated data further indicate that not only the listed spaces but also the key points occurring at these sites are highly relevant for the search for initial traces left by noxae (**Figures 2**-**7**; **Table 3**). A positional marker is the termination of the clear interface, which is located next to the head of the collecting duct (CD) ampulla and the distal end at the medial side of the arising pretubular aggregate (**Figures 2**, **3**). 1. Of special importance is the anchoring of the proximal end of the currently shaping nephron next to the connecting tubule of a previously formed nephron. 2. Equally meaningful is the temporary but partial separation of the renal vesicle from the residual pretubular aggregate (**Figures 3**-**5**). 3. Adjacent to this lies the largely unexplored transverse separation between the head (belonging to the district of progenitor cell recruitment) and the upper part of the conus of the CD ampulla (henceforth belonging to the area of nephron shaping). Damage to only one of these closely related key points may result in either developmental arrest or incorrect nephron formation.

Finally, future work should determine whether the condensate in the cap mesenchyme and the pretubular aggregate (**Figures 2**, **3**), positioned at the border between progenitor cell recruitment and nephron shaping, represent primary targets of noxae (62). Since a series of essential developmental processes are concentrated at this site (59, 60), blockage of this crucial region by noxae A, B, or C would be comparable to switching off the listed developmental key points 1, 2, and 3 (**Figure 2**). This remains a hypothesis; however, independent of the molecular nature of the noxae, the process of new nephron formation—including proximal and distal specialization—would come to a standstill at this location (66). The occurrence of essential structures within a very small spatial domain may also explain why noxae of diverse molecular composition ultimately produce the same outcome. The focus of future studies should be investigation of these aspects using a site-specific advanced culture model (67).

## 5 Conclusions

A series of quite different noxae can terminate formation of new nephrons in the fetal human kidney during advanced pregnancy. As a potential target, the border between progenitor cell recruitment and nephron shaping within the nephrogenic zone was investigated. The present microanatomical data show that this border runs along the condensate located at the far end of the cap mesenchyme and the distal end of the arising pretubular aggregate. At this location, the border coincides with termination of the clear interface separating the cap mesenchyme from the head of a CD ampulla. Adjacent to this site, adhesion of the future connecting tubule at the pretubular aggregate to the conus of the CD ampulla occurs. Thus, the inventory enables to focus on these developmental key points and detailed assessment of whether they represent central targets of noxae.

## Data Availability

The raw data supporting the conclusions of this article will be made available by the authors, without undue reservation.
